# Socio-Economic Burden of Influenza among Children Younger than 5 Years in the Outpatient Setting in Suzhou, China

**DOI:** 10.1371/journal.pone.0069035

**Published:** 2013-08-08

**Authors:** Dan Wang, Tao Zhang, Jing Wu, Yanwei Jiang, Yunfang Ding, Jun Hua, Ying Li, Jun Zhang, Liling Chen, Zijian Feng, Danielle Iuliano, Jeffrey McFarland, Genming Zhao

**Affiliations:** 1 Department of Epidemiology, School of Public Health, Fudan University, Key Laboratory of Public Health Safety, Ministry of Education, Shanghai, China; 2 Soochow University Affiliated Children's Hospital, Suzhou, China; 3 Suzhou Center for Disease Prevention and Control, Suzhou, China; 4 Chinese Center for Disease Control and Prevention, Beijing, China; 5 Centers for Disease Control and Prevention, Atlanta, Georgia, United States of America; Rosalind Franklin University of Medicine and Science, United States of America

## Abstract

**Background:**

The disease burden of children with laboratory-confirmed influenza in China has not been well described. The aim of this study was to understand the epidemiology and socio-economic burden of influenza in children younger than 5 years in outpatient and emergency department settings.

**Methods:**

A prospective study of laboratory-confirmed influenza among children presenting to the outpatient settings in Soochow University Affiliated Children's Hospital with symptoms of influenza-like illness (ILI) was performed from March 2011 to February 2012. Throat swabs were collected for detection of influenza virus by reverse transcription polymerase chain reaction assay. Data were collected using a researcher administered questionnaire, concerning demographics, clinical characteristics, direct and indirect costs, day care absence, parental work loss and similar respiratory illness development in the family.

**Results:**

Among a total of 6,901 children who sought care at internal outpatient settings, 1,726 (25%) fulfilled the criteria of ILI and 1,537 were enrolled. Influenza was documented in 365 (24%) of enrolled 1,537 ILI cases. Among positive patients, 52 (14%) were type A and 313 (86%) were type B. About 52% of influenza outpatients had over-the-counter medications before physician visit and 41% visited hospitals two or more times. Children who attended daycare missed an average of 1.9 days. For each child with influenza-confirmed disease, the parents missed a mean of 1.8 work days. Similar respiratory symptoms were reported in 43% of family contacts of influenza positive children after onset of the child's illness. The mean direct and indirect costs per episode of influenza were $123.4 for outpatient clinics and $134.6 for emergency departments, and $125.9 for influenza A and $127.5 for influenza B.

**Conclusions:**

Influenza is a common cause of influenza-like illness among children and has substantial socio-economic impact on children and their families regarding healthcare seeking and day care/work absence. The direct and indirect costs of childhood influenza impose a heavy financial burden on families. Prevention measures such as influenza vaccine could reduce the occurrence of influenza in children and the economic burden on families.

## Introduction

Seasonal influenza epidemics continue to cause substantial morbidity and mortality in children, generating increased burden on healthcare utilization. Children younger than 5 years have high rates of hospitalization attributable to influenza which are similar to or greater than rates among the elderly [1], [2], [3], [4]. A recent prospective surveillance study in three US counties showed an average of 0.9 per 1,000 children of 0–59 months of age was hospitalized with influenza [5]. Only 5% of children presenting to health clinics with influenza are hospitalized [6]. Most children with influenza are treated as outpatients, accounting for the greater part of the total disease burden in children [7]. The average annual rates of outpatient visits attributable to influenza were approximately 10, 100, and 250 times as high as hospitalization rates for children 0–5 months, 6–23 months, and 24–59 months of age, respectively [5]. Additionally, several studies have shown that young children are important in the transmission of influenza to their families and the broader community [6], [7], [8], [9]. Some data suggest that influenza immunization of young children can reduce disease rates in unimmunized members of the local community [10], [11]. The US Advisory Committee on Immunization Practices (ACIP) recommended that annual influenza vaccination be administrated to all children aged 6 months to18 years in 2009 [12].

Healthcare visits attributable to influenza virus infection among children can also impose substantial socio-economic burden on families and society. Where data are available, outpatient visits and hospitalizations due to influenza illness in children have been found to result in considerable medical expenses for their families; furthermore, the patients incur other non-medical costs including transportation, accommodation and additional nourishment. It was estimated that, in the U.S., the direct costs related to hospitalization due to influenza among children less than 5 years averaged $809.1 million annually, and the costs related to influenza-associated outpatient visits were $388.5 million [13]. In addition to the direct costs of medical care, influenza in this age group also has significant indirect costs especially in terms of parental work loss while caring for the sick child or because of secondary transmission of influenza [14], [15]. Estimates of the cost of influenza in USA, France and Germany have shown that indirect costs can be 5-to 10-fold higher than direct costs [16]. In light of the socio-economic burden, a variety of studies have assessed the potential cost-effectiveness of influenza vaccination of children, and find that vaccination is either cost saving or cost effective [17].

Despite extensive studies on disease burden of childhood influenza performed in developed countries and regions, the burden of influenza in East Asia, a region regarded as a common source of novel influenza epidemic and pandemic, has not been well described, especially in China, a country of 87 million 0–5 year old children. Influenza vaccination is not routinely recommended in China and immunization of children is uncommon [18]. Policy decisions regarding influenza vaccination in children require an accurate evaluation of the socio-economic burden of laboratory-confirmed influenza at the population level [19]. As such, the aim of this prospective study is to understand the epidemiological characteristics of laboratory-confirmed outpatient influenza in children younger than 5 years and comprehensively evaluate the socio-economic burden of childhood influenza on families and society, and provide evidence for decision makers to develop policy for influenza vaccination among children in China.

## Materials and Methods

### Study site

This study was conducted at Soochow University Affiliated Children's Hospital (SCH) in Jiangsu province, China. It is the single tertiary care children's hospital serving Suzhou district. An investigation on medical resources showed that in 2011 the total number of outpatient visits among children under 5 years at this hospital was 396,568 which accounted for approximately 28.5% of total amount of outpatient visits in municipal district of Suzhou (unpublished data).

### Study design

**Figure 1:**
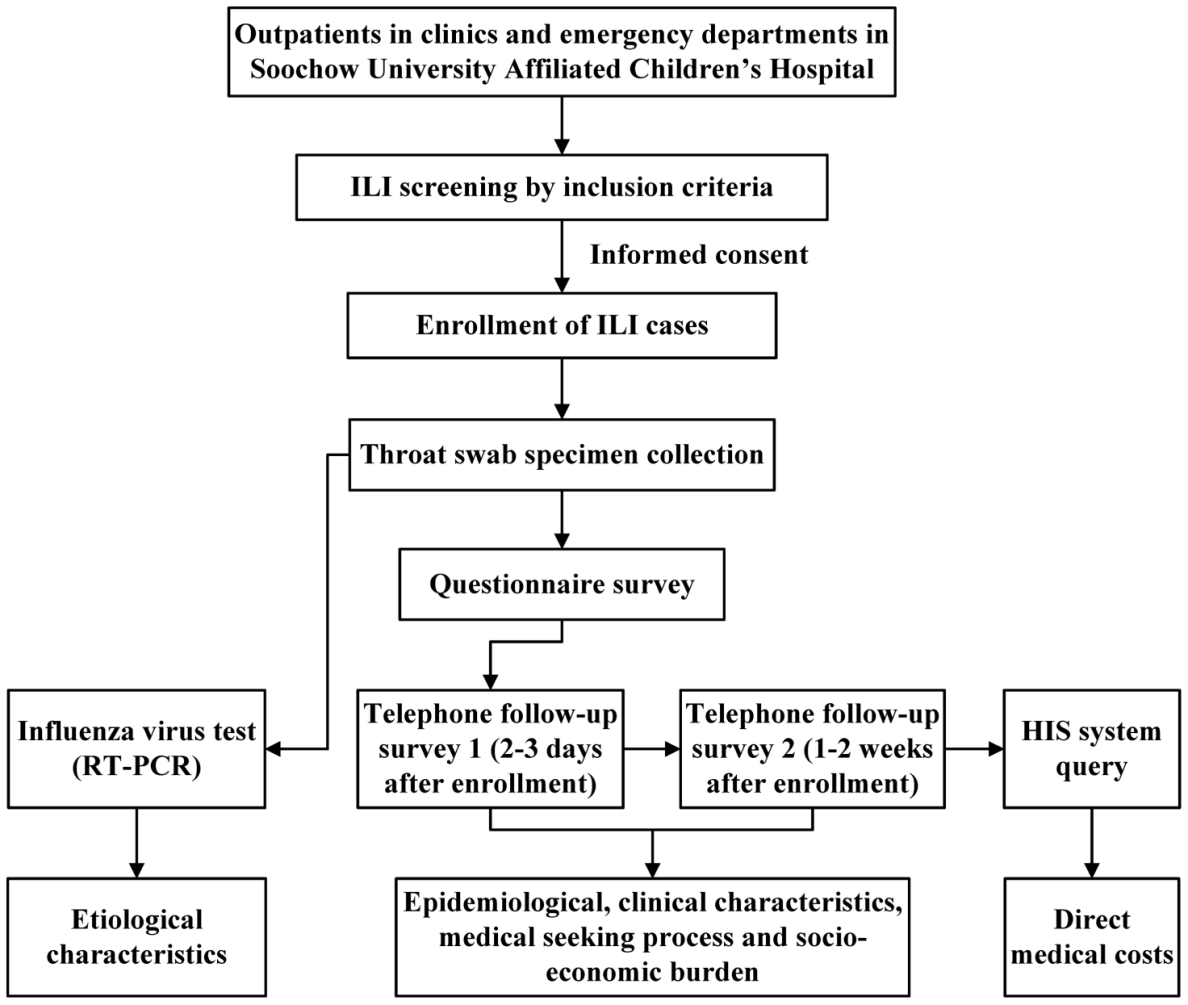
Study flowchart.

A prospective study of laboratory-confirmed influenza among children presenting to the outpatient settings in SCH with symptoms of influenza-like illness (ILI) was performed from March 2011 to February 2012.The study design is shown in Figure 1.

### Enrollment of patients

Children were enrolled in the surveillance if they were less than 5 years old and had symptoms of ILI defined as the presence of fever (axillary temperature≥38°C) and cough or sore throat (inflamed (red) pharynx was used to judge sore throat in young children), with onset of symptoms within the last 3 days. Enrollment of patients was performed five days per week in one to two clinics randomly selected from 20 internal outpatient clinics and internal emergency departments (ED). During the consultation time of the physician in the selected clinic, investigators assisted the physicians to screen children less than 5 years for ILI and record the total number of patients visited, those who fulfilled the criteria of ILI, the number of patients who were enrolled and sampled for influenza virus tests. Children who met the above criteria were recruited after obtaining the informed consent from their parents or guardians. Then investigators or physicians collected their throat swab specimen for influenza virus testing; and at the same time conducted a brief questionnaire survey to collect contact information, demographic data, past medical history, the course of illness, clinical manifestations, and medical seeking behaviors such as self-purchase of over-the-counter medicine and outpatient visits to other hospitals before enrollment.

### Follow-up survey and data collection

A follow-up telephone survey was administered to the parent or guardian of all enrolled ILI cases two times. During the first telephone survey, conducted within three days of patient enrollment, investigators called the parent or guardian to provide the results of influenza testing and collect information about the course of illness and medical treatment. One week later, investigators conducted a second telephone survey to collect data regarding the child's course of illness, medical seeking behaviors, number of outpatient visits, as well as expenses related to medical visits such as transportation, accommodation and additional nourishment. We also collected information about day care absence, parental work loss and similar illness development in family members after onset of the child's illness. If the loss of earnings could not be estimated by the parent, it would be calculated by multiplying the number of work days lost by the per capita daily income of 89.2 RMB for urban households and 44.1 RMB for rural households, obtained from the 2011 Suzhou statistical yearbook [20]. Transportation costs of driving a car would be converted into gas fees which were estimated by 1.5 RMB per kilometer or 60 RMB per hour's drive. In addition, the clinical diagnosis and direct medical costs of each enrolled patients were obtained by querying the Hospital Information System (HIS), which contains detailed line-item charges for registration, diagnostic tests, pharmaceuticals, therapeutics, etc. We used an exchange rate of 1 USD for 6.3 RMB to convert RMB costs to U.S. dollars.

### Specimen management and laboratory tests

Immediately after specimen collection, throat swabs were placed in tubes with viral transport media (Youkang Technology Co., Beijing, China) and stored in a refrigerator at −80°C or 4°C until transported. Study staffs were responsible for specimen transportation including maintaining the cold chain to the network laboratory of the National Influenza Surveillance Network of Suzhou CDC to detect influenza virus. The frequency of specimen transportation was about 2 to 3 times each week and influenza virus testing was completed the same day, both of which guaranteed timely laboratory tests and results feedback within 72 hours after collecting specimen. Viral RNA was extracted using High Pure Viral RNA Kit (Roche, Shanghai, China) according to the manufacturer's instructions. For influenza virus testing, we performed real-time reverse transcription polymerase chain reaction (rRT-PCR) using influenza virus A/B dual fluorescent quantitative RT-PCR kit (BioPerfectus Technology Co., Jiangsu, China). The influenza virus A subtype identification was performed using influenza virus A1/A3/A (H1N1) real time RT-PCR kit (ZJ Bio-Tech Co., Shanghai, China).

### Statistical analysis

Data analysis was performed using SPSS statistical package version 17.0 (SPSS Inc., Chicago, Illinois). Data were analyzed and compared using descriptive statistics. Categorical variables were presented as numbers or percentages, and compared by using Chi-square or two-tailed Fisher's exact test, whichever appropriate. Continuous variables were presented as the mean ± S.D. (standard deviation) or the mean with 95% confidence interval (CI) or as the median with inter-quartile range (IQR), and examined with student *t* test or nonparametric test.

### Ethics statement

The study was approved by the Institute Review Board (IRB) of School of Public Health, Fudan University. Written informed consent was obtained from parents or guardians on behalf of children participants involved in the study before specimen collection and questionnaire survey.

## Results

### Characteristics of study participants

From March 2011 to February 2012, we examined a total of 6,901 children less than five years who sought care at internal outpatient clinics and ED in SCH mainly for respiratory illness. Of those, 1,726 (25.0%) fulfilled the criteria of ILI. Among the ILI cases, 1,537 (89.0%) were enrolled in our study. Among the enrolled ILI cases, 1,005 (65.4%) were from outpatient clinics, and 532 (34.6%) from ED. The male to female ratio was 1.3∶1, and the median age was 21.4 months (IQR: 11.1–37.9). Young children less than 1 year accounted for a higher proportion of patients from ED than those from outpatient clinics (30.3% vs. 23.4%, *P*<0.05). Underlying medical conditions were present more often in patients from the ED compared with those from outpatient clinics (7.4% vs. 4.3%, *P*<0.05). There were 899 (58.5%) ILI cases that had medical insurance (Table 1).

**Table 1:** Characteristics of enrolled influenza-like illness (ILI) cases, by medical setting and influenza virus status, n (%).

Characteristics	Medical setting		Influenza virus		Total (N = 1537)
	Outpatient clinics (N = 1005)	ED (N = 532)	Positive (N = 365)	Negative (N = 1172)	
Gender					
Male	565 (56.2)	302 (56.8)	213 (58.4)	654 (55.8)	867 (56.4)
Female	440 (43.8)	230 (43.2)	152 (41.6)	518 (44.2)	670 (43.6)
Age^c^^,^ d					
0–5 m	42 (4.2)	35 (6.6)	14 (3.8)	63 (5.4)	77 (5.0)
6–11 m	193 (19.2)	126 (23.7)	33 (9.0)	286 (24.4)	319 (20.8)
12–23 m	286 (28.5)	147 (27.6)	84 (23.0)	349 (29.8)	433 (28.2)
24–35 m	172 (17.1)	91 (17.1)	82 (22.5)	181 (15.4)	263 (17.1)
36–47 m	156 (15.5)	79 (14.8)	61 (16.7)	174 (14.8)	235 (15.3)
48–59 m	156 (15.5)	54 (10.2)	91 (24.9)	119 (10.2)	210 (13.7)
District^a^					
Municipal district	817 (84.1)	441 (85.5)	300 (85.5)	958 (84.3)	1258 (84.6)
Suburban county	154 (15.9)	75 (14.5)	51 (14.5)	178 (15.7)	229 (15.4)
Medical insurance					
Yes	595 (59.2)	304 (57.1)	198 (54.2)	701 (59.8)	899 (58.5)
No	410 (40.8)	228 (42.9)	167 (45.8)	471 (40.2)	638 (41.5)
Total family income prior year (thousand U.S. dollars)					
<15.9	462 (57.7)	206 (57.7)	164 (55.8)	504 (58.3)	668 (57.7)
15.9–23.8	213 (26.6)	74 (20.7)	67 (22.8)	220 (25.5)	287 (24.8)
23.8–31.7	74 (9.2)	44 (12.3)	33 (11.2)	85 (9.8)	118 (10.2)
31.7–39.7	25 (3.1)	14 (3.9)	13 (4.4)	26 (3.0)	39 (3.4)
>39.7	27 (3.4)	19 (5.3)	17 (5.8)	29 (3.4)	46 (4.0)
Underlying medical conditions^b^^,^ c	41/948 (4.3)	38/512 (7.4)	19/340 (5.6)	60/1120 (5.4)	79/1460 (5.4)

Note:

a Municipal district refers to seven central urban districts and suburban county refers to five county-level districts in Suzhou;

b Underlying medical conditions include asthma, chronic pulmonary disease, congenital heart disease, neuromuscular disease, renal dysfunction, blood disorders, immunosuppression, etc;

c Chi-square *P*<0.05 for comparison between ILI cases from outpatient clinics and ED;

d Chi-square *P*<0.05 for comparison between influenza positive and negative ILI cases.

### Epidemiological characteristics

**Figure 2:**
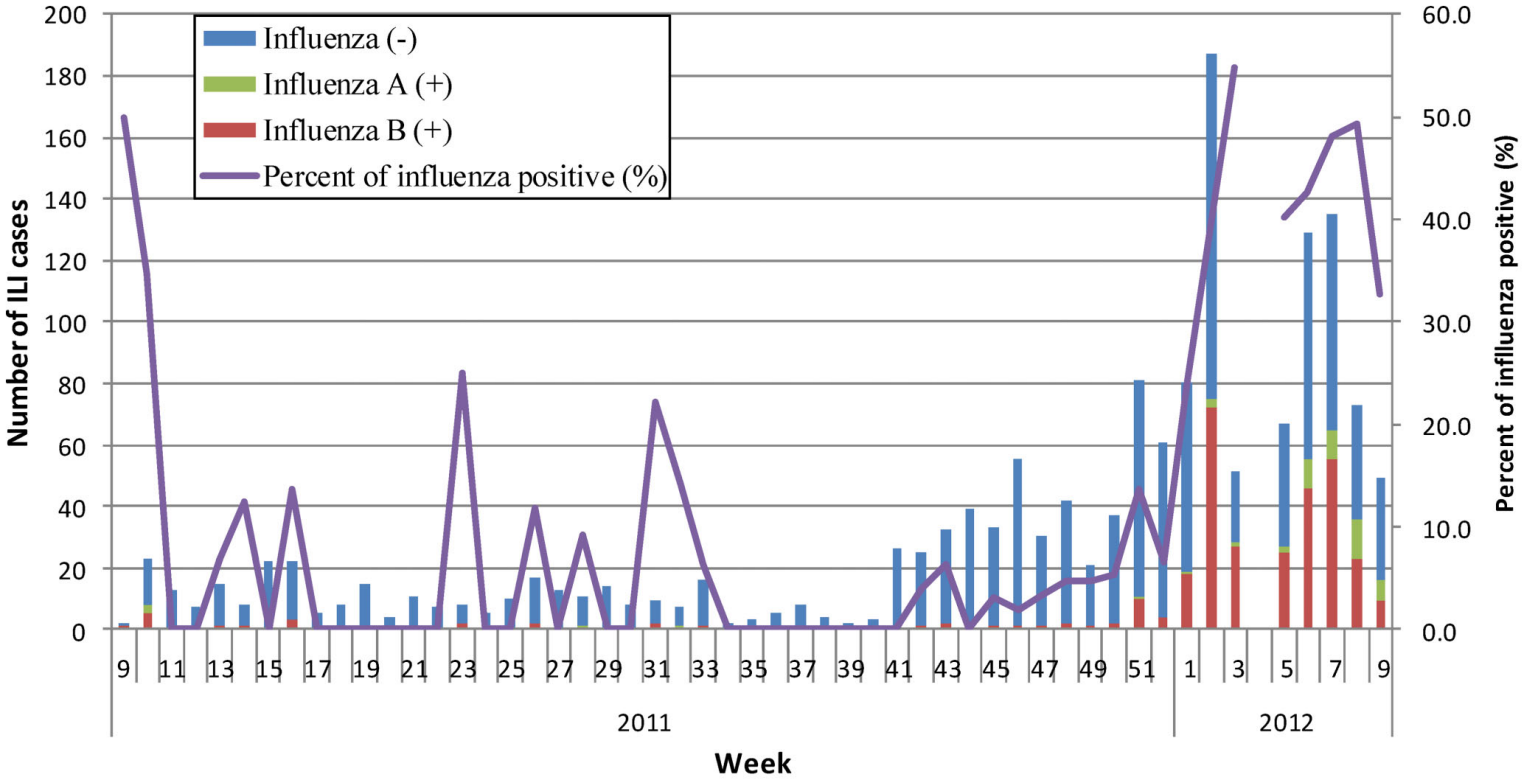
Distribution of enrolled influenza-like illness (ILI) cases and laboratory confirmed influenza cases in Soochow University Affiliated Children's Hospital (SCH) between March 2011 and February 2012. Note: No specimens were collected during week 4 of 2012 due to the Spring Festival; the subtype for all influenza A viruses was seasonal H3.

All of the 1,537 ILI cases were tested for influenza virus, with 23.7% (365) of them positive for influenza virus. Among the positive cases, 52 (14.2%) were positive for influenza A (H3N2) and 313 (85.8%) positive for influenza B. The distribution of influenza positive cases formed an evident epidemic from December 2011 to February 2012, with influenza B predominating. The peak influenza percent positive (at least 10% of the total number of positive influenza virus tests in any two consecutive weeks) was between the second and ninth weeks in 2012, and the highest influenza percent positive (54.9%) occurred in the third week of 2012 (Figure 2).

### Characteristics of influenza positive cases

Of 365 influenza positive cases, 241 cases were from outpatient clinics and 124 from ED. The median age was 31.9 months (IQR: 16.8–47.5), with a male to female ratio of 1.4∶1. Three hundred (85.5%) patients were from the municipal district and 51(14.5%) from the suburban county. One hundred ninety-eight (54.2%) patients were covered by medical insurance (on average, medical insurance covered 10% of the cost of care for respiratory illness). Underlying medical conditions were present in 5.6% of influenza positive cases. In comparison with influenza negative cases, there was a higher proportion of children aged 25–60 months that were influenza positive (61.6% vs. 37.8%, *P*<0.001, Table 1). The characteristics of influenza positive cases were similar between patients from outpatient clinics and ED and between patients with influenza A and B.

Besides fever, the most frequent symptoms of influenza positive cases were cough (70.4%) and rhinorrhea (24.9%). The mean highest measured temperature was higher in patients from ED (39.2±0.4°C) than those from outpatient clinics (38.7±0.6°C). The mean duration of symptoms before enrollment was 1.9±1.0 days. Bronchitis (17.4%) and pneumonia (17.1%) were the most common diagnoses of influenza infection, followed by sinusitis (2.9%), asthma (2.3%), croup (2.6%) and febrile seizures (1.1%). There were no significant differences regarding clinical characteristics between patients from outpatient clinics and ED and between patients with influenza A and B.

### Medical seeking behaviors of influenza positive cases

There were 51.8% of influenza positive cases that had over-the-counter medications before seeking care at an outpatient clinic. More influenza positive children from the suburban county had visited hospitals other than SCH during the illness, compared with those from the municipal district (50.0% vs. 31.2%, *P*<0.05). No significant difference was found between influenza positive and negative cases regarding medical seeking behaviors prior to visiting SCH (Table 2).

**Table 2:** Medical seeking behaviors of influenza-like illness (ILI) cases, by influenza virus status and district, n (%).

	Influenza (+) (N = 350)			Influenza (−) (N = 1135)	*P* c
	Municipal district (N = 299)	Suburban county (N = 51)	Total (N = 350)		
Over-the-counter medications	154/290 (53.1)	22/50 (44.0)	176/340 (51.8)	508/1111 (45.7)	0.069
Visits to hospitals other than SCH^a^ , b	91/292 (31.2)	25/50 (50.0)	116/342 (33.9)	347/1111 (31.2)	0.442
Number of outpatient visits in SCH^b^					0.015
1	170 (56.9)	37 (72.5)	207 (59.1)	626 (55.2)	
2	91 (30.4)	11 (21.6)	102 (29.1)	301 (26.5)	
≥3	38 (12.7)	3 (5.9)	41 (11.7)	208 (18.3)	

Note:

a Soochow University Affiliated Children's Hospital (SCH);

b Chi-square *P*<0.05 for comparison between two districts among influenza positive cases;

c Chi-square *P* value for comparison between influenza positive and negative cases.

In addition, we analyzed the total number of clinic visits to SCH for each ILI cases. We counted the number of clinic visits within a one month period since the child's onset of illness. The child was considered cured if no treatment was given for 2 consecutive weeks. Of 1,537 ILI cases, the number of clinic visits was unobtainable in 52 cases from either HIS or telephone follow-up. Among the remaining 1,485 ILI cases, the mean number of clinic visits in influenza positive cases was 1.6±1.0, which was slightly lower than that of influenza negative cases (1.7±1.0) (*P* = 0.054). Among influenza positive cases, the mean number of clinic visits for influenza cases from municipal district was significantly more than those from suburban county (1.7±1.0 vs. 1.3±0.6, *P*<0.05). More children from municipal district than those from suburban county had visited SCH two or more times during the illness (42.9% vs. 27.5%, *P*<0.05, Table 2).

### Economic burden of influenza

A direct medical cost survey was completed in 1,528 ILI cases (99.4%) by queries to HIS. Information about non-medical costs and indirect medical costs were collected by follow-up telephone survey and was completed by the parent or guardian of 1,341 ILI cases (86.7%); the remaining 196 were either lost to follow-up or the patient's family member refused to participate in the additional survey. In total, 1,333 ILI cases were included in the non-medical costs and indirect costs analysis.

The average direct medical cost per episode of influenza was $63.1 (95%CI, 58.1–68.0), including $61.6 (95%CI, 56.0–67.1) for outpatient clinics and $66.0 (95%CI, 56.2–75.7) for ED; the cost was $59.1 (95%CI, 48.8–69.4) for influenza A and $63.7 (95%CI, 58.2–69.3) for influenza B. Pharmaceuticals accounted for the majority of the outpatient costs (73.4%), followed by laboratory tests (11.4%), therapeutics (2.5%), materials (2.3%), radiology (2.2%), and registration (1.9%). Taking the direct non-medical costs such as transportation, accommodation and additional nourishment into consideration, the mean total direct costs per episode of influenza was $88.2 (95%CI, 80.9–95.6). The breakdown of direct costs was similar when stratified by medical setting and viral type. Compared with influenza negative cases, pharmacies and radiology fees were higher in influenza positive cases, while laboratory tests, therapeutics and additional nourishment fees were lower (*P*<0.05). In addition, influenza illness also incurred substantial indirect costs, typically because of lost earnings due to parental work absence. For each influenza-confirmed child, the parents missed a mean of 1.8±2.6 work days to care for the sick child, resulting in average lost earnings of $38.7 (95%CI, 30.3–47.2), which was not significantly different compared with influenza negative cases (Table 3).

**Table 3:** Direct and indirect costs per episode of influenza-like illness (ILI) for influenza positive and negative cases, by medical setting and viral type among influenza positive cases, mean U.S. dollars (95%CI)^a^.

	Influenza virus status		Medical setting^e^		Influenza viral type^e^	
	Influenza (+)	Influenza (−)	Outpatient clinics	ED	Influenza A	Influenza B
**Direct medical costs**						
Registration	1.9 (1.7–2.1)	2.0 (1.9–2.2)	1.8 (1.6–2.1)	2.0 (1.6–2.4)	1.7 (1.4–2.1)	1.9 (1.7–2.2)
Materials	2.3 (1.8–2.8)	2.2 (1.9–2.4)	2.3 (1.7–3.0)	2.3 (1.3–3.1)	2.2 (0.7–3.7)	2.3 (1.8–2.9)
Radiology	2.2 (1.6–2.9)^d^	1.7 (1.4–2.0)	2.1 (1.4–2.9)	2.4 (1.1–3.7)	2.3 (0.2–4.5)	2.2 (1.5–2.9)
Laboratory test	7.2 (6.6–7.8)^d^	8.1 (7.7–8.6)	6.3 (5.7–6.9)	8.8 (7.4–10.2)	6.4 (5.1–7.7)	7.3 (6.6–8.0)
Pharmaceuticals	46.3 (42.5–50.2)^d^	43.7 (41.5–45.9)	46.1 (41.7–50.6)	46.8 (39.6–54.0)	43.3 (35.3–51.3)	46.9 (42.6–51.1)
Therapeutics	2.5 (2.2–2.9)^d^	3.3 (3.0–3.6)	2.4 (2.0–2.9)	2.7 (2.0–3.3)	2.8 (1.7–3.9)	2.5 (2.1–2.8)
Others^b^	0.3 (0.1–0.4)	0.3 (0.3–0.4)	0.2 (0–0.3)	0.5 (0.2–0.7)	0.3 (0.1–0.6)	0.3 (0.1–0.4)
Subtotal	63.1 (58.1–68.0)	61.6 (58.8–64.4)	61.6 (56.0–67.1)	66.0 (56.2–75.7)	59.1 (48.8–69.4)	63.7 (58.2–69.3)
**Direct non-medical costs**						
Transportation	21.4 (18.2–24.7)	21.4 (19.7–23.2)	19.6 (16.2–23.0)	25.1 (18.0–32.2)	21.0 (13.9–28.1)	21.6 (17.9–25.3)
Additional nourishment	2.2 (0.7–3.7)^d^	5.6 (4.3–7.0)	2.3 (0.6–4.0)	2.3 (0–5.3)	2.5 (0.3–4.8)	2.2 (0.5–3.9)
Others^c^	2.0 (1.6–2.5)	1.9 (1.4–2.5)	2.1 (1.5–2.8)	1.8 (1.1–2.5)	3.0 (1.3–4.6)	1.9 (1.4–2.4)
Subtotal	26.6 (22.5–30.6)	29.1 (26.7–31.6)	24.0 (20.0–27.9)	30.7 (21.7–39.7)	26.5 (18.7–34.3)	26.6 (22.0–31.1)
**Total direct costs**	88.2 (80.9–95.6)	93.4 (88.7–98.1)	85.1 (77.2–93.0)	94.2 (79.0–109.5)	83.7 (67.8–99.7)	89.0 (80.8–97.2)
**Indirect costs**						
Parental loss of earnings	38.7 (30.3–47.2)	34.9 (30.7–39.2)	37.8 (28.0–47.5)	41.0 (24.1–57.9)	42.2 (26.5–57.8)	38.3 (28.6–48.0)
**Total direct and indirect costs**	127.2 (115.1–139.4)	128.1 (121.3–134.9)	123.4 (109.6–137.3)	134.6 (110.8–158.3)	125.9 (100.2–151.6)	127.5 (113.9–141.1)

Note:

a We used an exchange rate of 1 USD for 6.3 RMB to convert RMB costs to U.S. dollars;

b Others include bed, nursing, air-condition fees, etc;

c Others include expenses of accommodation, toy, etc;

d *P*<0.05 in comparison between influenza positive and negative cases;

e Comparison among influenza positive cases by medical setting and viral type.

These results enabled us to calculate the mean total direct and indirect costs for one episode of influenza. The mean total direct and indirect cost was $123.4 (95%CI, 109.6–137.3) for outpatient clinics and $134.6 (95%CI, 110.8–158.3) for ED, and $125.9 (95%CI, 100.2–151.6) for influenza A and $127.5 (95%CI, 113.9–141.1) for influenza B (Table 3).

### Other impacts on sick children and their families

Among influenza positive children older than 3 years, 33.1% had daycare absence due to their illness and missed a mean of 1.9±3.1 days. However, influenza negative children had a larger proportion of daycare absence (46.1%) and missed significantly more days (2.9±4.4 days) than influenza positive children (*P*<0.05). In addition to parental work loss to care for the sick child, self-reported respiratory symptoms occurring after onset of their child's illness were more common in the family members of influenza positive children than in family members of influenza negative children (42.7% vs. 29.5%, *P*<0.001). Of family members who self-reported a respiratory illness, 90.3% of family members of influenza positive children purchased over-the-counter medicine or had a healthcare visit for their own illness, a higher percent than among family members of influenza negative children (83.2%) (Table 4).

**Table 4:** Socio-economic impacts of influenza-like illness (ILI) on children and their families, by influenza virus status.

	Influenza virus status		*P* c
	Influenza (+)	Influenza (−)	
Absence from day care, n (%)^a^	45/136 (33.1)	118/256 (46.1)	0.013
Lost day care days, mean±S.D.	1.9±3.1	2.9±4.4	0.018
Lost parental workdays, mean±S.D.	1.8±2.6	1.7±2.4	0.400
Similar respiratory symptoms in family members, n (%)	144/337 (42.7)	325/775 (29.5)	<0.001
Over-the-counter medication or healthcare seeking, n (%)^b^	130/144 (90.3)	267/321 (83.2)	0.045

Note:

a Day care absence was evaluated among children≥3 years who had attended day care;

b Over-the-counter medication and healthcare seeking due to similar respiratory symptoms developed in family members;

c Chi-square *P* value for comparison between influenza positive and negative cases.

## Discussion

In this prospective study, we comprehensively described the socio-economic burden of laboratory-confirmed influenza of a sample of young children treated as outpatients in one district in mainland China. As the single tertiary care children's hospital serving Suzhou district, SCH accounted for 30% of total outpatient visits among children under 5 years old in the municipal district of Suzhou. Our surveillance data showed a substantial level of influenza activity among young children who sought care during March 2011–February 2012. The influenza season began in the 2^nd^ week 2012, showing an evident winter-spring seasonal peak with influenza B predominating. The seasonal pattern was consistent with reports from provinces in southern China reported in the National Influenza Sentinel Surveillance Weekly Reports [21]. The overall influenza percent positive for the study period (23.7%) was consistent with that reported from the nearby city of Shanghai in the outpatient setting during the preceding two years 2009–2011 (25.8%) [22].

The majority of influenza positive children for our study population were 24–59 months of age. This may be explained by the higher proportion of children attending daycare in this age group. Numerous studies have suggested that children in daycare facilities are at greater risk for respiratory infections than are children cared for at home [23], [24]. Therefore, the implementation of immunization with an effective influenza vaccine in children of this age group may have a considerable impact in the reduction of influenza-associated morbidity [6].

Children are also important vectors for the transmission of influenza within families and communities [25], [26]. In our study, the family contacts of influenza positive children self-reported similar respiratory symptoms after onset of their child's illness more frequently than those of influenza negative children, and required more over-the-counter medicine or clinic visits, in accordance with recent studies [6], [7], [8], [9], [15], [19]. Although influenza was not laboratory confirmed in family contacts, our results reflected a higher risk of developing ILI symptoms among caretakers of children with influenza, which might increase the cost of medical care for the family. Further studies, including laboratory confirmation of influenza or other respiratory viruses among family contacts, are needed to confirm these findings and to better understand household transmission in this population.

Healthcare utilization attributable to influenza illness in children directly imposed medical expenses on families. It was found in our study that the average direct medical cost per episode of influenza was $63.1, which was much higher than Guo [27] reported ($22.8) in a study of all age groups in Zhuhai City in South China. However, our results from two medical settings were much lower than those from the US, $167 per influenza-associated outpatient visit in 2003 [13] and $512 per influenza-associated ED visit in 2003–2006 [28]. These disparities may be attributable to the differing healthcare systems and average income levels of the two countries; even inside China the economic differences are obvious between different regions. Our medical costs did not include costs associated with medical seeking behaviors other than outpatient visits to SCH. As we reported, 51.8% of children with influenza had taken over-the-counter medicine. Additionally, many of the children enrolled, especially those from suburban districts, had additional healthcare visits to other hospital facilities prior to seeking care at SCH. The lack of data on medical costs incurred from these health visits probably resulted in an underestimate of our direct medical costs.

In addition to medical costs, our study also documented other non-medical expenses that were incurred during the process of healthcare seeking, such as transportation, accommodation and additional nourishment costs. The mean transportation cost in our study was $21.4 per episode of influenza, constituting the majority of non-medical costs, being much higher than that from US and Thailand [23], [29]. It is likely to be influenced by medical care seeking behavior. As a single tertiary children's hospital serving Suzhou district, SCH was a priority hospital for most parents of children when their child had to see a doctor, regardless of the distance from their home [30]. It can be demonstrated that 14.5% of visits of children with influenza were from remote suburban counties, thus a large amount of money would be spent on transportation costs. Further investigations to better understand all components of the costs associated with utilizing healthcare services are needed to support these findings.

Influenza infection in children also had significant indirect costs, for example parental work absence and the resulting loss of productivity. Several studies have shown that indirect costs accounted for the majority of the total costs [15], [29], [31], [32]. In our study, parents missed a mean of 1.8 work days to care for the influenza infected child, resulting in average lost earnings of $38.7. The average loss of wages was approximately one-third of the total cost for medically attended influenza in outpatient settings. Our results are slightly lower compared with reports from other countries [15], [33]. It is important to note that the time lost from work in our study may be underestimated because it did not include the medical costs and workdays lost due to possible secondary transmission of influenza in the family. Therefore, it is reasonable to assume that the actual indirect costs may be even higher.

The socio-economic burden of childhood influenza on families was high in our study, with the mean total cost of both direct and indirect expenses being $127.2 for one episode of influenza. This accounts for approximately 30% and 60% of the average monthly household income in 2011 for local urban and rural residents, respectively [20]. Furthermore, these are only estimates of medically-attended influenza in outpatient settings since our study was designed to evaluate the burden of influenza in one hospital's outpatient department and only included the cost associated with the visit to our study site. Influenza-associated hospitalizations as well as seeking care at multiple facilities would likely result in greater socio-economic burden.

In many countries, influenza B is considered to play a less important role in influenza disease burden [15], [34]. However, in our study, the influenza B was the most predominant strain that circulated among our influenza-like illness cases, which is consistent with the circulating influenza viruses in south China during the same time. In addition, the socio-economic burden of influenza B cases was slightly higher than that of influenza A cases (*p*>0.05). Further, some studies have reported that the influenza B patients experienced more severe illness than seasonal influenza A patients [35], which may contribute to the higher cost of influenza B virus infection. Disease burden and cost associated with medically attended influenza virus infection vary from year to year, and may be influenced by circulating viral strains [14]. Thus, studies for continuous years are needed to evaluate the disease and economic burden of influenza.

Some limitations to our study should be noted. Firstly, we did not test for other respiratory pathogens, specifically respiratory syncytial virus (RSV), which often represents a confounding variable in influenza research as it has similar symptoms and seasonal patterns [36]. It is necessary to further understand the etiological spectrum to differentiate the contribution of other pathogens. Secondly, our results are from only one tertiary hospital, which may not be representative of the entire Suzhou district. Patients who visit tertiary hospitals are usually more severely ill than those who visit other outpatient clinics. However, our previous investigation on health services utilization showed that more than half of the parents living in Suzhou municipal district prefer to take their child to SCH for medical care if their child developed symptoms of ILI. Thirdly, the follow-up surveys were self-reported and may be subject to recall bias regarding costs and illness questions.

In spite of these limitations, our study provided a comprehensive evaluation of the socio-economic burden of pediatric influenza in China, which will provide data that can be used by other regions in China to estimate the disease burden of influenza as well as by health policy-makers to consider the potential benefits of implementing influenza vaccination in children.

## Conclusions

Influenza virus infection occurred in approximately one-quarter of cases of ILI and has substantial socio-economic impact on children and their families regarding healthcare seeking and day care/work absence. The direct and indirect costs of childhood influenza in the outpatient setting impose a heavy financial burden on families in China. Prevention measures such as influenza vaccine could reduce the occurrence of influenza in children and reduce the economic burden on families.
